# Antibacterial Effect of Shrimp By-Products Hydrolysate on Specific Spoilage Organisms of Squid

**DOI:** 10.3390/molecules28104105

**Published:** 2023-05-15

**Authors:** Luo Gu, Qiuyu Zhu, Xiaoyu Zou, Ru Song

**Affiliations:** Key Laboratory of Health Risk Factors for Seafood of Zhejiang Province, School of Food Science and Pharmacy, Zhejiang Ocean University, Zhoushan 316022, China; s20083200021@zjou.edu.cn (L.G.); zhuqiuyu@zjou.edu.cn (Q.Z.); zouxy_job@163.com (X.Z.)

**Keywords:** shrimp processing by-products, pepsin hydrolysis, squid, specific spoilage organisms, antibacterial effect, bacterial diversity

## Abstract

In order to further develop and utilize shrimp processing by-products, in this study, a novel antibacterial hydrolysate of shrimp by-products by pepsin hydrolysis (SPH) was prepared. The antibacterial effect of SPH on specific spoilage organisms of squid after end storage at room temperature (SE–SSOs) was investigated. SPH showed an antibacterial effect on the growth of SE–SSOs, with (23.4 ± 0.2) mm of inhibition zone diameter. The cell permeability of SE–SSOs was enhanced after SPH treatment for 12 h. Some bacteria were twisted and shrunk, while pits and pores formed and intracellular contents leaked under scanning electron microscopy observation. The flora diversity of SE–SSOs treated with SPH was determined by a 16S rDNA sequencing technique. Results showed that SE–SSOs were mainly composed of the phyla of Firmicutes and Proteobacteria, among which *Paraclostridium* (47.29%) and *Enterobacter* (38.35%) were dominant genera. SPH treatment resulted in a significant reduction in the relative abundance of the genus *Paraclostridium* and increased the abundance of *Enterococcus*. Linear discriminant analysis (LDA) of LEfSe conveyed that SPH treatment had a significant impact on altering the bacterial structure of SE–SSOs. The 16S PICRUSt of Cluster of Orthologous Group (COG) annotation revealed that SPH treatment for 12 h could significantly increase the function of transcription level [K], while SPH treatment for 24 h could downregulate post-translational modifications, protein turnover, and chaperone metabolism functions [O]. In conclusion, SPH has a proper antibacterial effect on SE–SSOs and can change the flora structure of SE–SSOs. These findings will provide a technical basis for the development of inhibitors of squid SSOs.

## 1. Introduction

Penaeus sinensis (*Solenocera crassicornis*), which are commonly known as red shrimp and have a body length of 5–10 cm, can be captured in the south of Yellow Sea, the East China Sea, South China Sea, and the adjacent waters of Southeast Asia [1]. It is one of the main shrimp species that is processed in China. During shrimp processing, 50–60% of shrimp by-products, including heads, shells, and tails, are produced [2]. These by-products also contain nutritional ingredients, including lipids, proteins, amino acids, astaxanthin, etc. [3,4,5]. In recent years, the development of various by-products as natural preservatives to increase food safety has attracted great attention. These processes include the extraction of grape seeds with chitosan edible coating film to extend the shelf life of refrigerated rainbow trout fillet [6], as well as the extraction of color from the red onion skin against the growth of *Staphylococcus aureus* and *Escherichia coli* [7]. Therefore, the rational utilization of by-products from shrimp processing can not only reduce the waste of resources and harm to the ecological environment but also will considerably increase the added value of these by-products.

Protease hydrolysis (endogenous enzymes or added commercial proteases) can hydrolyze proteins from aquatic products to release bioactive peptides. In recent years, the preparation of antibacterial peptides or antibacterial hydrolysate by enzymatic hydrolysis has attracted great attention. For example, the enzymatic hydrolysate from Tilapia fish waste showed different antibacterial effects on *Staphylococcus aureus*, *Bacillus subtilis*, and *Escherichia coli* [8]. Peptide fractions with a molecular weight of 200–750 Da were isolated from the enzymatic hydrolysate of by-products of Atlantic rock crab, which showed strong antibacterial effects on spoilage bacteria and pathogenic bacteria such as *Aeromonas hydrophila*, *Campylobacter jejuni*, and *Listeria monocytogenes* [9]. The peptides cleaved from hemocyanin with antibacterial, and antifungal activities were found in the shrimp *Fenneropenaeus chinensis* [10]. Peptide extracts from the hydrolysates of white shrimp (*Litopenaeus vannamei*) by-products exhibited antibacterial activities against *Yersinia ruckeri*, *Bacillus megaterium*, and *Edwardsiella tarda* [11]. The enzymatic preparation of antibacterial peptides or antibacterial hydrolysates has the advantages of mild reaction conditions and easy control of the reaction process. Therefore, antibacterial hydrolysates or peptides prepared by protease hydrolysis could be an ideal approach for mass production of antibacterial agents [12].

Squid is an important deep-sea fishery resource with a moisture content as high as 70–85%, so it is more prone to microbial spoilage than general land animal meat. The spoilage process of aquatic products is initially involved in a variety of microorganisms, but by the time the spoilage stage is reached, only a few microorganisms have an absolute advantage in the density of bacteria. These bacteria, which cause the spoilage of aquatic products and are dominate in density, are called specific spoilage organisms (SSOs) [13]. In recent years, with the rapid development of Miseq sequencing technology, 16S rDNA amplicon sequencing has been widely used for the analysis of the structural composition of microbial communities and the identification of characteristic bacteria in the hypervariable region of aquatic products, such as the analysis of microbial diversity during the storage of large yellow croaker (*Pseudosciaena crocea*) [14], common carp (*Cyprinus carpio*) [15], manila clam (*Ruditapes philippinarum*) [16], and Antarctic krill (*Euphausia superba*) [17]. The use of biological agents with high safety can delay the spoilage of squid to a certain extent. For example, propolis can slow down the rate of browning and oxidation of squid during storage and significantly reduce the total bacterial count [18]. Karim et al. (2016) reported that squid coated with 0.025% squid ink can effectively delay squid spoilage and extend shelf life [19]. In addition, ε-polylysine hydrochloride and thymol were effective in inhibiting squid microbial growth and muscle lipid oxidation, slowing down meat spoilage and deterioration [20]. However, there are few reports on the preparation of antibacterial hydrolysates from shrimp processing by-products to inhibit squid spoilage bacteria.

In this study, five protein hydrolysates were prepared by the digestion of shrimp by-products, and their antibacterial effects on specific spoilage organisms of squid at the end of storage (SE–SSOs) at room temperature were compared. Among which, the most antibacterial hydrolysate was selected to further determine its antibacterial activity on SE–SSOs by measuring the membrane permeability and integrity. Furthermore, the effect of this antibacterial hydrolysate selected on the diversity of SE-SSOs was evaluated using16S rDNA sequencing technique. All these findings will provide evidence for the use of by-products from shrimp by-products in the development of antibacterial agents for squid preservation.

## 2. Results and Discussion

### 2.1. Comparison of Antibacterial Activities among Five Hydrolysates

**Figure 1:**
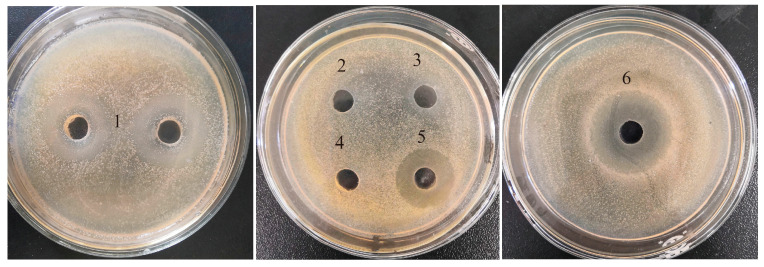
Comparison of inhibition zone of five different hydrolysates against SE–SSOs by agar-well diffusion method. Nos. 1—blank control; 2—neutral protease hydrolysate; 3—flavourzyme hydrolysate; 4—trypsin hydrolysate; 5—alcalase hydrolysate; 6—pepsin hydrolysate. The diameter of inhibition zone was measured by a vernier caliper.

Bioactive peptides hidden in their parent protein sequences can be released by enzymatic hydrolysis. Some commercial enzymes, such as neutral protease, alcalase, and pepsin (which are characterized by their high specificities for hydrophobic amino acids); trypsin with specific cleavage sites for peptide bonds formed by arginine or lysine residue; and flavourzyme with high hydrolysis degree for proteins have been employed in the production of antibacterial hydrolysates [21,22,23,24]. However, the appropriate proteolytic enzymes selected may be varied due to the different hydrolysis materials or hydrolysis conditions used. In order to obtain the most antibacterial hydrolysates from the shrimp by-product, in this study, five commercial proteases, including neutral protease, flavourzyme, trypsin, alcalase, and pepsin, were used to hydrolysis shrimp by-products at their respective suitable hydrolysis pH and temperatures (see Table 1). In addition, their antibacterial activities against SE–SSOs were compared by the agar-well diffusion method (Figure 1).

Figure 1 shows that both pepsin and alcalase hydrolysates of shrimp by-products demonstrated a bacteriostatic effect on SE–SSOs, with the diameters of inhibition zones of 23.4 ± 0.2 mm for pepsin hydrolysate and 16.5 ± 0.5 mm for alcalase hydrolysate, respectively. By comparison, the other four hydrolysates showed weak or less antibacterial activities on SE–SSOs. Similar to our results, the half-fin anchovy pepsin hydrolysates had stronger antibacterial effects than other hydrolysates [21]. Hydrolysate from trout by-products using trout pepsin showed good inhibitory activities against *Flavobacterium psychrophilum* and *Salmoninarum renibacterium* [25]. Pepsin has a specific ability to digest peptide bonds formed with the participation of hydrophobic amino acids, such as phenylalanine, leucine, tryptophan, and tyrosine, and to produce peptides with hydrophobic amino acids at the N- or C-terminal [26], which could contribute to the solubility of peptides in aqueous environments and further enter into lipid-rich membranes to target antibacterial sites [27]. Our findings revealed that antibacterial stretches hidden in shrimp by-product proteins can be released through pepsin hydrolysis.

### 2.2. Effects of Hydrolysis Conditions on Antibacterial Activity of Pepsin Hydrolysate on SE-SSOs

**Figure 2:**
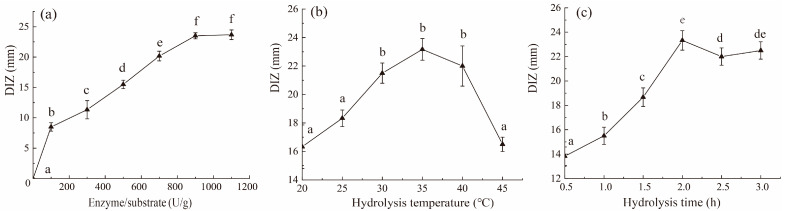
Effects of pepsin hydrolysis conditions on the antibacterial activity of shrimp by-products hydrolysate against SE–SSOs. (**a**) Amount of added pepsin; (**b**) hydrolysis temperature; (**c**) hydrolysis time. Under different hydrolysis conditions (enzyme/substrate, hydrolysis temperature, and hydrolysis time), the significant differences in DIZ between samples were represented by different lowercase letters (*n* = 3) (*p* < 0.05).

According to Figure 1, pepsin was selected as the most suitable enzyme to prepare antibacterial hydrolysate from shrimp by-products. The effects of added pepsin content, hydrolysis temperature, and hydrolysis time on the diameter of the inhibition zone (DIZ) of SE–SSOs were further evaluated, as shown in Figure 2.

The bacteriostatic effect of pepsin hydrolysate on SE–SSOs increased with the increase of pepsin addition (Figure 2a). No significant difference was found for the DIZ between pepsin addition of 900 U/g and 1100 U/g (*p* < 0.05). This meant that an amount of more than 900 U/g could meet the requirements for pepsin used in hydrolysis. A higher amount of pepsin will not only increase the preparation cost but also might further digest the antibacterial peptide fractions released, which might cause a decrease or loss of antibacterial activity.

When 900 U/g of pepsin was added, the DIZ of pepsin hydrolysates prepared at a hydrolysis temperature of 30–40 °C were significantly larger than those of other hydrolysis temperatures (*p* < 0.05) (Figure 2b). Low temperatures are not conducive to pepsin activity, whereas high temperatures could easily inactivate pepsin, which might explain the lower DIZ of pepsin hydrolysate generated at lower or higher hydrolysis temperatures [21].

The antibacterial effect of pepsin hydrolysate on SE–SSOs increased with the prolongation of hydrolysis time (Figure 2c). After 2 h of hydrolysis, the DIZ of pepsin hydrolysate reached a high level, then decreased with the increase of hydrolysis time. Pepsin hydrolysis that is sustained for long amounts of time can lead to hydrophobic amino acid residues releasing from generated hydrophobic peptides, while the antibacterial activity of free hydrophobic amino acids is generally lower than that of hydrophobic peptides [21,26,27]. Therefore, we speculate that the decrease in antibacterial activity of pepsin hydrolysate of shrimp processing by-products after more than 2 h of hydrolysis should be related to the degradation of antibacterial peptidic fractions.

Considering the data from Figure 1 and Figure 2, the antibacterial hydrolysate of shrimp processing by-products by pepsin was prepared at pH 2.0, with 900 U/g of pepsin added and hydrolyzed at 35 °C for 2 h. The generated antibacterial hydrolysate was named SPH, which showed clear DIZ against SE–SSOs (Figure S1).

### 2.3. Molecular Weight (Mw) Distribution of Peptide Fractions in SPH

**Figure 3:**
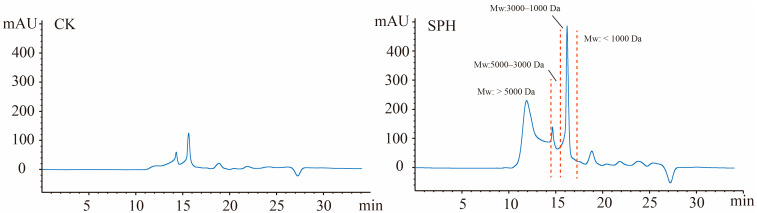
The molecular weight distribution of peptide fractions in SPH by high performance liquid chromatography on a PL aquagel–OH 30 column (7.5 × 300 mm, 8 μm) detected at 220 nm. CK-Undigested shrimp by-products; SPH- shrimp by-products hydrolysate by pepsin digestion under optimum conditions.

The peptide concentration of SPH was 36.39 mg/mL. Compared with undigested shrimp by-products (CK), obvious peptide fractions were detected in SPH, suggesting large amounts of peptides were released after pepsin digestion (Figure 3). Among these, four peptide fractions with Mw of >5000 Da, 5000–3000 Da, 3000–1000 Da, and <1000 Da were 48.01%, 9.51%, 25.97%, and 16.60%, respectively. A peptide fraction with Mw > 5000 Da should contain pepsin because during the preparation of SPH, after thermal inactivation and centrifugation, some pepsin was inevitably contained in the SPH solution. In addition to the peak with Mw greater than 5000 Da, smaller peptides with Mw of 2000 to 350 Da should be dominant in SPH. Similarly, Robert described that most of hydrolytic peptides from the antibacterial hydrolysate of white shrimp had a MW below 2000 Da [11]. Short antibacterial peptides with MWs below 1000 Da were identified from the pepsin hydrolysate of *Limnospira maxima* [28]. Our findings suggested that the by-products of shrimp processing hydrolyzed by pepsin can be used to produce smaller peptides with potential antibacterial effects.

### 2.4. Antibacterial Effect of SPH on SE–SSOs

As an important site for bacterial metabolic activities, cell membranes can control the exchange and penetration of substances inside and outside the cell. Therefore, they can regulate the balance between the bacteria and the external environment [29]. Once the integrity of the cell membrane is damaged, some low-molecular-weight substances, such as K^+^ and PO_4_^3−^, often leak, while DNA, RNA, and other substances soon leak out of the cell as well [21]. Antibacterial hydrolysates or peptides could have the capacity to disrupt plasma membranes, thus leading to membrane permeabilization and nucleotide leakage [21,30]. These intracellular leakage substances have a specific absorbance at 260 nm. Therefore, the change of the absorbance at 260 nm is often used to indicate the extent of cell membrane damage [31].

**Figure 4:**
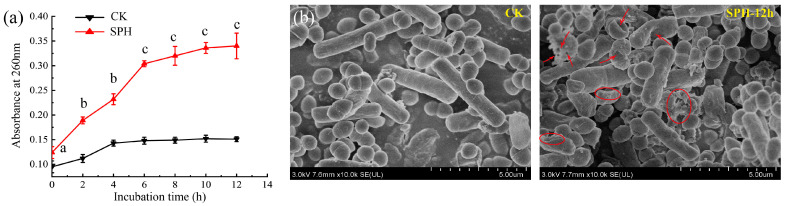
Effects of SPH on the cell integrity and microstructure changes of SE–SSOs. (**a**) Cell membrane permeability was measured as a release of intracellular components at 260 nm, CK– SE–SSOs without SPH treatment; SPH– SE–SSOs treated by SPH; and (**b**) Micromorphological changes were observed under scanning electron microscopy (SEM) after incubation for 12 h with SPH. At different incubation times, the significant differences in absorbance of the same group of samples at 260 nm were represented by different lowercase letters (*n* = 3) (*p* < 0.05). The formation of pits and pores on SE–SSOs were indicated with red arrows. The irregularity and roughness of SE–SSOs were marked with red circles.

As was shown in Figure 4a, the absorbance of SE–SSOs treated by SPH at 260 nm increased, and a sharp increase was observed from 0 h to 6 h. By comparison, the absorbance slightly changed in the CK group. These findings indicated that SPH treatment caused membrane disruption in SE–SSOs to some extent, resulting in intracellular substance leakage. When the action lasted for 12 h, the absorbance of the SPH-treated group did not significantly increase, which might be related to the thorough leakage of K^+^, PO_4_^3−^, DNA, and RNA l in the damaged cells.

To further understand the antibacterial effect of SPH on SE–SSOs, the microstructural changes of SE–SSOs after 12 h of SPH treatment were observed by SEM. The presence of *bacilli* and *cocci* in SE–SSOs revealed that there was more than one dominant spoilage bacteria in SE–SSOs. These SSOs in the CK group remained intact and smooth without apparent abnormalities and disruption of cell membranes (Figure 4b). In contrast, under the action of SPH, the morphology and cell structure of SE–SSOs were changed. The membrane surface of some bacteria became irregular and rough, with wrinkles, concave, and ghost-like appearances. Moreover, holes were observed in some cells, resulting in the spillage of cell contents. All results elucidated that SPH treatment increased the membrane permeabilization of SE–SSOs and caused the leakage of intracellular components to different degrees. The microstructural changes of SE–SSOs observed in Figure 4b were consistent with the increased leakage of intracellular components in Figure 4a. Therefore, it is supposed that SPH may achieve the antibacterial effect on SE–SSOs by destroying bacterial cell integrity via irreversible cell membrane damage.

### 2.5. Effect of SPH on Microbial Structure of SE–SSOs

#### 2.5.1. Alpha Diversity

The indices of Ace, Chao1, Shannon, and Simpson are usually used to evaluate the microflora diversity [32,33]. In this study, after 12 h or 24 h of SPH treatment, the changes of alpha diversity of SE–SSOs were determined (Table 2).

The coverage index of each group reached above 0.999, indicating a high coverage rate of the sample library and a low probability of undetected sequences. Therefore, these detected sequences in this study can be used for microbial diversity analysis. Among these indices related to alpha diversity, Ace and Chao1 indices represent microbial abundance; and the higher the value, the greater the microbial abundance. (Shannon and Simpson indices reflect community diversity, for example. Greater Shannon and lower Simpson indices are generally associated with higher microbial diversity [32].) Compared with the CK group, there was no significant difference in the Ace and Chao1 indices in the SPH-treated groups (SPH–12 h and SPH–24 h) (*p* > 0.05). However, the Ace and Chao1 indices in the SPH–24 h group were significantly higher than those in the SPH–12 h group (*p* < 0.05), suggesting the bacterial abundance increased in the SPH–24 h group. After SPH treatment, some bacteria in SE–SSOs underwent bacterial rupture, as shown in Figure 4, and the substances leaking might have a certain promoting effect on the growth and reproduction of other SPH tolerant bacteria in SE–SSOs. Therefore, after the enrichment and cultivation of SE–SSOs treated with SPH (as described in Section 3.8 for bacterial DNA extraction), a large number of SPH-tolerant bacteria proliferated. Due to the longer 24-h treatment time, the rupture of some bacteria in SE–SSOs could become more severe, leading to more growth-promoting factors being released in the SPH–24 h treatment group. This might explain a significantly higher abundance of bacteria in the SPH–24 h group than in the SPH–12 h group. However, the Shannon index of SPH–12 h or SPH–24 h group was significantly lower than that of the CK group (*p* < 0.05), which meant SPH treatment significantly reduced the bacterial diversity in SE–SSOs. Similarly, higher Simpson indices of the SPH–12 h and SPH–24 h groups also suggested microbial diversity reduction in SE–SSOs after SPH treatment.

#### 2.5.2. Beta Diversity

**Figure 5:**
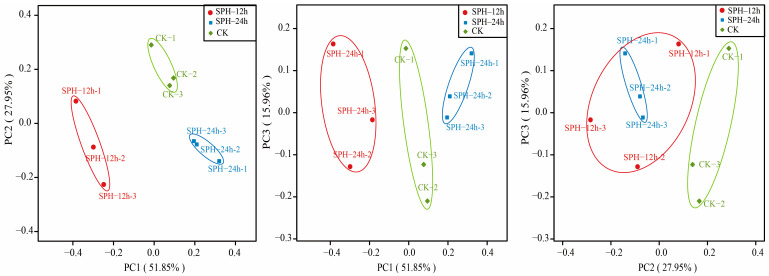
Principal coordinate analysis (PCoA) distribution of SE–SSOs after SPH treatment for 12 h and 24 h compared to the CK.

Beta diversity can evaluate the differences between microbial communities among different groups. PCoA can distinguish the similarity of specimens at the genus level. The more similar the sample composition is, the closer the points are on the PCoA diagram [33]. In Figure 5, PC1, PC2, and PC3 accounted for 51.85%, 27.95%, and 15.96% of the variation, respectively (more than 95% in total). This meant that the original sample information can be well explained. Distinguished clusters between the CK- and SPH-treated groups (SPH–12 h and SPH–24 h) revealed that SPH treatment resulted in changes in bacterial structure in SE–SSOs at the genus level. The observed overlap of specimens between the SPH–12 h and SPH–24 h groups also indicated their similarities to some degree at the genus level.

#### 2.5.3. Composition of SE–SSOs at the Phylum, Family, and Genus Levels

**Figure 6:**
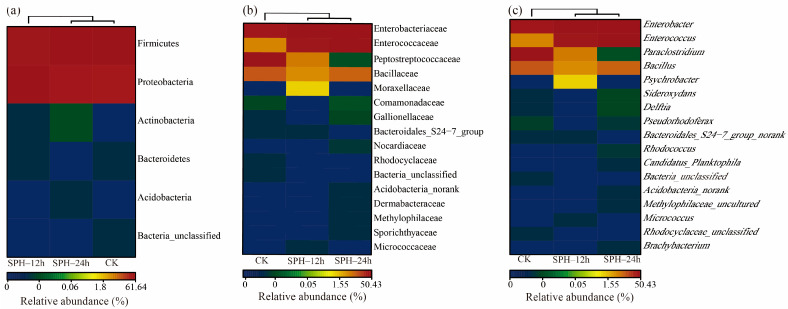
Comparison of bacterial composition of SE–SSOs at the (**a**) phylum, (**b**) family, and (**c**) genus levels after SPH treatment for 12 h and 24 h compared to the CK.

At the phylum level (Figure 6a), firmicutes and proteobacteria were two abundant phyla, accounting for >99.9% of the total sequences. After SPH treatment, the relative abundance of firmicutes in SE–SSOs (61.64%) decreased to 48.51% in SPH–12 h and 57.24% in SPH–24 h. In contrast, after 12 h of SPH treatment, the relative abundance of proteobacteria in SE–SSOs (38.36%) increased to 51.49% in SPH–12 h and 42.75% in SPH–24 h. These findings suggested that SPH should have a certain effect on altering the proportion of dominant spoilage bacteria in SE–SSOs. Similar to our results, an increase of proteobacteria abundance in grass carp treated with ε-poly-lysine gradually increased with storage time, while the relative abundance of firmicutes and other phyla decreased [34].

At the family level (Figure 6b), SE–SSOs were mainly composed of four families in the phylum of Firmicutes, including Peptostreptococcaceae (47.29%), Enterobacteriaceae (38.35%), Bacillaceae (9.88%), and Enterococcaceae (4.47%). After SPH treatment, the relative abundance of conditional pathogen Peptostreptococcaceae in SE–SSOs decreased to 5.34% in SPH–12 h and less than 0.001% in SPH–24 h, respectively. Compared with the CK group, the relative abundance of the Enterobacteriaceae family was improved slightly in the SPH–12 h and SPH–24 h groups. The relative abundance of Bacillacea decreased to 3.93% in SPH–12 h. A slight increase of Bacillacea in SPH–24 h (8.45%) might be related to the increased tolerance of Bacillaceae due to long action time. It was noted that the relative abundance of Enterococcaceae family in SPH–12 h and SPH–24 h, was enhanced to 39.24% and 48.79%, respectively, compared with that of 4.47% in the CK group (*p* < 0.05). Enterococcaceae is a group of gram-positive facultative anaerobic coccus, which has a broad application in the food industry used as probiotics [35]. This result indicated the contribution of SPH treatment in reducing the harmful bacteria rate and increasing the proportion of potential probiotics in comparison.

At the genus level (Figure 6c), SE–SSOs were mainly composed of *Paraclostridium* (47.29%), *Enterobacter* (38.35%), *Bacillus* (9.88%), and *Enterococcus* (4.47%). By comparing the relative abundance of the above bacteria, it was found that their relative abundances were consistent with those abundances at the family level, indicating that SE–SSOs were mainly concentrated on the two genera *Paraclostridium* (belonging to Peptostreptococcaceae family, Firmicute phylum) and *Enterobacter* (belonging to Enterobacteriaceae family, Proteobacteria phylum), accounting for >85% in total bacteria. SPH–12 h and SPH–24 h groups had lower relative abundances in *Paraclostridium* than the CK group. According to documents, the genus of *Paraclostridium* was detected in anaerobic spoilage bacteria from beef carcasses [36], but this type of bacterium has not been reported in spoilage aquatic products. It was speculated that some factors, such as the living environment, fishing season, and storage conditions of aquatic products, might be responsible for this result. Although the relative abundance of *Enterobacter* in CK, SPH–12 h and SPH–24 h surpassed 35%, no significant differences were found among the three groups (*p* < 0.05). This meant that SPH exhibited an inhibitory effect on specific bacteria in SE–SSOs except for the genus *Enterobacter*, which was consistent with SEM results. This means some bacteria still maintained complete bacterial structure (as shown in Figure 3b for SPH–12 h). In contrast, after 12 h and 24 h of SPH treatment, the relative abundance of *Enterococcus* in SE–SSOs was significantly higher than the CK group. Our findings revealed the altering effect of SPH on the bacteria structure of SE–SSOs by inhibiting or enhancing specific bacteria growth and reproduction.

### 2.6. LEfSe–Linear Discriminant Analysis (LDA)

**Figure 7:**
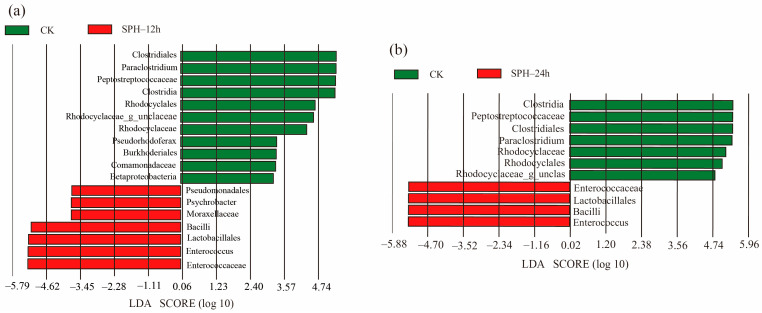
LEfSe–LDA of significant bacteria between the CK and SPH–12 h groups (**a**), between the CK and SPH–24 h groups (**b**).

To further distinguish specific bacteria responsible for the differences between the CK group *vis* SPH–12 h group and the CK group *vis* SPH–24 h group, the LEfSe–LDA (LDA score > 2) was performed, as shown in Figure 7.

The genus of *Paraclostridium,* belonging to the Peptostreptococcaceae family, the Clostridiales order, and the Clostridia class, was responsible for distinguishing the CK group from the SPH–12 h group (*p* < 0.05) (Figure 7a). In addition, the genus of *Rhodocyclaceae_g_unclaceae*, belonging to the Rhodocyclales family and Rhodocyclaceae order, and the *Betaproteobacteria* species, belonging to the *Pseudorhodoferax* genus, as well as the Burkholderiale order and the Comamonadaceae family, made contributions to separate the CK group from the SPH–12 h group. By comparison, the genera of *Psychrobacter* belonging to the Moraxellaceae family, the Pseudomonadales order, and the genus *Enterococcus* belonging to the Enterococcaceae family, Lactobacillales order, and Bacilli class were all identified as specific bacteria with high contributions to distinguish the SPH–12 h group from the CK group.

Similarly, the genera of *Paraclostridium* and *Rhodocyclaceae_g_unclaceae* in the CK group, and the genus of *Enterococcus* in SPH–24 h, were all specific bacteria to distinguish the two groups (Figure 7b). *Psychrobacter* species might enhance food spoilage during aerobic storage under cold conditions by degrading lipids, amino acids, and proteins [37]. The disappearance of *Psychrobacter* genus in the SPH–24 h group might be related to the inhibition of growth and reproduction by long-term incubation at 37 °C.

Our findings conveyed that two dominant genera, *Paraclostridium* in the CK group and *Enterococcus* in the SPH-treated group, could be responsible for the bacterial structure differences between SE–SSOs and SPH treatments. According to documents, some species of the genus *Paraclostridium* were proposed with pathogenic features, such as *Paraclostridium dentum* [38] and *Paraclostridium bifermentans* [39]. Decreases of the abundance of genus *Paraclostridium* in SPH–12 h or SPH–24 h compared to the CK group conveyed the good inhibitory effect of SPH on potential pathogenic *Paraclostridium* in SE–SSOs.

According to the literature, the genus *Enterococcus* comprises more than 20 species and plays a double effect in the food industry [40]. It can be used as a starter or probiotic culture in food preservation by inhibiting the growth of certain pathogenic and spoilage microorganisms [41]. At the same time, virulence factors in food are also related to several species of *Enterococci*, such as *Enterococcus faecium* and *Enterococcus faecalis*, the two most prevalent *Enterococci* in foods related to human pathology [42,43]. In this study, due to the depth limitation of 16S sequencing, the species in *Enterococcus* were identified as *Enterococcus_unclassified*. Therefore, subsequent experiments need to characterize possible species of *Enterococci* that might exist in SE–SSOs after SPH treatment.

### 2.7. Changes of Gene Functions of SE–SSOs after SPH Treatment

**Figure 8:**
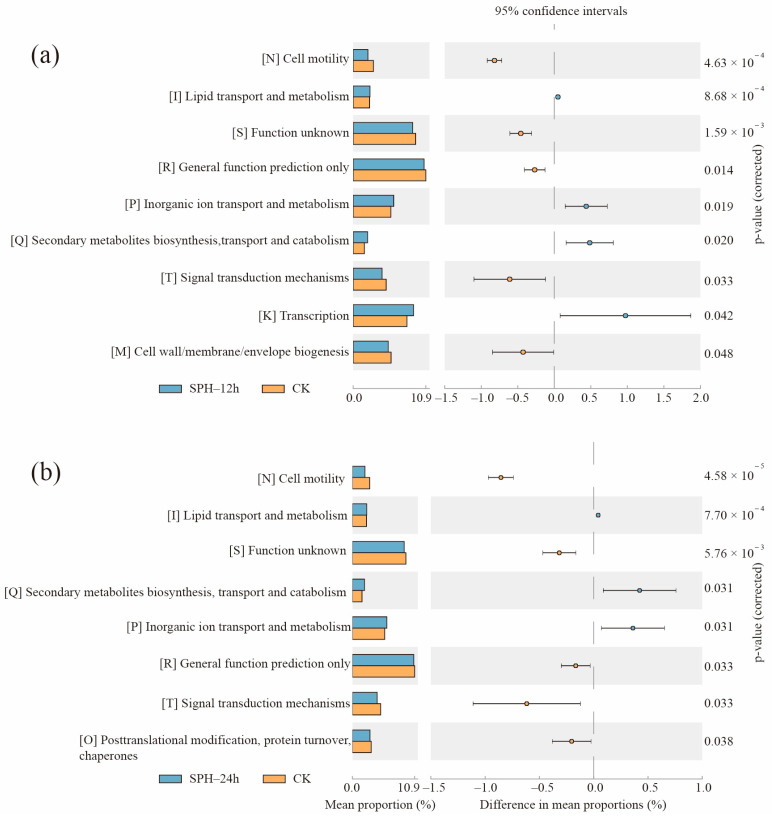
Predicting functional differences between the CK and SPH–12h groups (**a**), the CK and SPH–24h groups (**b**) using Picrut COG annotations.

After SPH treatment for 12 h or 24 h, the 16S phylogenetic investigation of communities by the reconstruction of unobserved states (PICRUSt) of Cluster of Orthologous Group (COG) was used to annotate the differential gene functions of SE–SSOs (Figure 8).

Compared with the CK group, nine functions in the SPH–12 h group (Figure 8a) and eight functions in the SPH–24 h group (Figure 8b) changed significantly (*p* < 0.05). Among them, the cell motility [N], function unknown [S], and signal transduction mechanisms [T] in both SPH–12 h and SPH–24 h groups were dramatically lower than those in the CK group, while the lipid transport and metabolism [I], inorganic ion transport and metabolism [P], and secondary metabolite biosynthesis, transport, and catabolism [Q] were all significantly higher than those in the CK group.

Compared to the CK group, SPH–12 h significantly reduced the biological formation of the cell wall/membrane/envelope biogenesis [M] (Figure 8a), suggesting less biofilm formation in SE–SSOs after SPH treatment. Biofilm is a source of cross-contamination in food, which can lead to the resistance of spoilage microorganisms to chemical preservatives, thereby reducing the effectiveness of food processing strategies and damaging food quality and safety [44]. Some species identified in the genus *Paraclostridium* could form biofilm [38] and decrease the relative abundance of the genus *Paraclostridium* in the SPH–12 h or SPH–24 h groups (as shown in Figure 6c). Therefore, this indicates a reduction of biofilm formation to some extent after SPH treatment. Antibacterial peptides can eradicate biofilm, as Zhang et al. (2021) reported three natural antimicrobial peptides (As–CATH4, As–CATH5, and Hc–CATH) that demonstrated anti-biofilm properties on spoilage bacteria [45]. In this study, the decrease of biofilm formation predicted in the SPH–12 h group might be related to the inhibitory effect of antibacterial peptides in SPH on the reproduction of specific bacteria with biofilm formation. In contrast, the increase of transcription [K] level in the SPH–12 h group implied that the upregulation of [I], [P], and [Q] functions might be associated with the increased mRNA type and abundance of SE–SSOs after 12 h of SPH treatment. It is considered that modification of proteins by post-translational modifications (PTMs) acts as a powerful mechanism to reversibly diversify the functions of proteins [46]. Compared with the CK group, the function of posttranslational modification, protein turnover, and chaperones [O] (including PTMs) were all downregulated in the SPH–24 h group (Figure 8b). This meant the functional diversity of proteins of SE–SSOs could be blocked after SPH treatment for 24 h.

Based on the above results, we speculate that decreases of [N] and [T] functions, as well as [M] in SPH–12 h and [O] in SPH–24 h, might be ascribed to the antibacterial activity of SPH against some genera in SE–SSOs, such as *Paraclostridium*, while increases of *Enterococcus* strain reproduction in SPH–12 h and SPH–24 h groups might be one of the reasons for enhancing [I], [P], and [Q] functions. At the present study, the peptide sequence responsible for the inhibitory effect of SPH on SE–SSOs has not been elucidated. However, our findings confirm the antibacterial activity of SPH against specific genera in SE–SSOs. The targeting pathways of SPH on specific bacteria in SE–SSOs need to be further explored.

## 3. Materials and Methods

### 3.1. Materials

Penaeus sinensis (*Solenocera crassicornis*) and squid were purchased from a local aquatic products market (Zhoushan, China). Neutral protease (50,000 U/g), flavourzyme (15,000 U/g), alkaline protease (20,000 U/g), and trypsin (250 U/mg) were obtained from Solarbio Technology Co., Ltd. (Beijing, China). Pepsin (1200 U/g) was provided by Sinopharm Chemical Reagent Co., Ltd. (Shanghai, China). Bovine serum albumin (BSA, 68,000 Da), Vitamin B_12_ (1355.37 Da), oxidized glutathione (GSSG, 612.63 Da), and reduced glutathione (GSH, 307.32 Da) were obtained from Aladdin (Shanghai, China). Nutrient agar and peptone were bought form Qingdao Haibo Microbial Technology Co., Ltd. (Qingdao, China). All other reagents used in this study were of analytical grade and were commercially available.

### 3.2. Preparation of Protein Hydrolysates from Shrimp Processing By-Products

Five different protein hydrolysates of shrimp processing by-products were prepared according to method of Song et al. (2012) [21], with slight modifications. In brief, the shrimp by-products, including heads and shells, were minced into uniformity sludge with a breaker (HH–200C, Cixi Kandun Huihao Electrical Appliance Co., Ltd., Cixi, China). Then, deionized water was added at a ratio of 2:1 (*v*/*w*) into the sludge in a beaker (250 mL) and blurred completely. The pH of the mixture was adjusted to the optimal values (shown in Table 1) using 6 mol/L HCl or 6 mol/L NaOH. The hydrolysis reaction was started by the addition of enzyme at the same activity level (enzyme/substrate, 900 U/g) to compare hydrolysis efficiency. The amounts of protease added to the reaction were calculated based on the substrate (shrimp by-product weight, in grams). The pH of the mixture was maintained constant for 2 h using 6 mol/L HCl or 6 mol/L NaOH during the reaction. After hydrolysis, the mixture was heated at 100 °C for 10 min to inactivate the enzymes, and the pH was adjusted to 7, followed by centrifugation at 8000× *g* for 20 min at 4 °C (Heraeus Multifuge X1R High-speed centrifuge, Hunan Xiangyi Instrument Co., Ltd., Xiangtan, China). The supernatant was collected, divided, and stored at −20 °C for later use. The supernatant without enzymatic hydrolysis was used as blank control.

### 3.3. SE–SSOs Collection

Fresh squid were cut into pieces and placed in sterile Petri dishes at room temperature until severe spoilage occurred (about 6 d). The squid pieces were washed with a small amount of sterile water, and the bacterial suspension collected were named as SE–SSOs. SE–SSOs were subpackaged into sterile centrifuge tubes for further use.

### 3.4. Antibacterial Activity—Agar-Well-Diffusion Method

The antibacterial activities of shrimp by-product hydrolysates prepared in 3.2 were compared according to method of agar-well diffusion [21], with further modifications. In brief, SE–SSOs were cultured into log phase in liquid nutrient broth (about 18–24 h with 0.40 of absorbance at 600 nm), pipetted out 100 μL of suspension into a sterilized Petri dish, followed by 18 mL of nutrient agar (45–50 °C) addition and blending. A sterilized stainless-steel punch (10-mm diameter) was used to obtain wells after the agar became solid. Then, 150 μL of hydrolysates were added to each well. The plate was incubated at 37 °C for 24 h, and the DIZ around the well was measured.

Based on the result of agar-well diffusion, pepsin was selected as an optimum enzyme. The effects of additional pepsin content (0–1200 U/g), hydrolysis temperature (20–45 °C), and hydrolysis time (0.5–3.0 h) on the antibacterial activity were further investigated to determine appropriate hydrolysis conditions for preparation of SPH, filtrated with sterilized 0.22-μm filter membrane to remove bacteria in next experiment.

### 3.5. Molecular Weight Distribution

Peptide concentration in SPH was determined by o–phthaldialdehyde method [47] and expressed as mg/mL. The molecular weight distribution of peptide fractions in SPH was analyzed by high-performance liquid chromatography (HPLC) system (Agilent 1260 Infinity, Waldbronn, Germany) according to our previous study [48], with slight modifications. In brief, 10 μL of SPH were loaded on a PL aquagel–OH 30 column (7.5 × 300 mm, 8 μm) at 20 °C. The mobile phase was 30% methanol (containing 0.1% of formic acid) at flow rate of 0.5 mL/min, and the online detection wavelength was 220 nm. Mw standards, including BSA (Mw: 68,000 Da), Vitamin B_12_ (Mw: 1355.37 Da), GSSG (Mw: 612.03Da), and GSH (Mw: 307.32 Da), were detected under the same conditions. The elution time (min) was used as the abscission (x), and the logarithm of relative Mw (lg Mt) of standards was used as the ordinate (y) to obtain the Mw calibration curve (y = −0.2713x + 7.6762, R^2^ = 0.9998). The Mw of peptide fractions in SPH was determined according to the Mw standard curve. The relative percentage (%) of peptide fractions, with Mw > 5000 Da, 5000–3000 Da, 3000–1000 Da, and <1000 Da, were determined by comparing each peptide fraction area with the total peak area.

### 3.6. Membrane Permeability

SPH was blended with SE–SSOs in logarithmic phase at a ratio of 1:1 (*V*:*V*). During incubation at 37 °C for 12 h, 1 mL of the mixture was pipetted out every 2 h and centrifuged at 3500× *g* for 10 min. The supernatant collected was filtered through 0.22-μm micro-membrane to further remove residue bacteria. After diluted appropriately with 0.9% NaCl, the absorbance of supernatant at 260 nm was measured using a TU–1810PC UV–Vis spectrophotometer (Beijing, China). The higher the absorbance value, the greater the leakage of intracellular genetic ingredients [21]. SPH itself is a peptide mixture with strong absorption at 260 nm. In this study, an equal proportion of SPH diluent was used as a zero-adjustment tube to determine the absorbance of SE–SSOs at 260 nm after SPH treatment.

### 3.7. Microstructure Observation—SEM

Bacterial sediments of SE–SSOs were collected after 12 h of treatment by SPH described in 3.6, carefully washed at least twice with sterile saline, then fixed overnight (4 °C) in 2.5% glutaraldehyde. The fixed bacteria were dried by the critical point drying method. After sprayed with gold, the microstructure of bacteria was observed using a Hitachi SU8020 (Japan) field emission-scanning electron microscope at an accelerating voltage of 3.0 kV. SE–SSOs without SPH treatment in 3.6 under the same conditions were used as a control group.

### 3.8. 16S rDNA Sequencing and Bioinformatics Analysis

Bacterial DNA extraction and amplification: SE–SSOs in logarithmic phase were blended with SPH at a volume ratio of 1:1 (*V*:*V*) and incubated at 37 °C for 12 and 24 h, respectively. Then, 50 μL of mixture were pipetted out and inoculated into 10 mL of sterile liquid nutriment broth to accumulation culture for 24 h at 37 °C, followed by centrifugation at 3500× *g* for 10 min. The bacterial precipitates collected were used for total DNA extraction, according to the operating instructions of bacterial DNA extraction kit. The polymerase chain reaction (PCR) amplification of bacterial 16S rDNA was performed at Origingene (Shanghai, China) with a specific barcode primer 515F_907R(515F, 5′-GTGCCAGCMGCCGCGG-3′;907R, 5′-CCGTCAATTCMTTTRAGTTT-3′) as follows: 95 °C for 3 min, 27 cycles (95 °C for 30 s, 55 °C for 30s, and 72 °C for 45 s), and 72 °C for 5 min, maintained at 10 °C until stopped. The concentration of PCR products was determined by Qubit 3.0 fluorometer (Thermo Scientific, Carlsbad, CA, USA). The quality of PCR amplification was measured by 2% agarose gel electrophoresis, and the AxyPrep DNA gel recovery kit (AXYGEN Company, Union City, CA, USA) was used to cut and recover the PCR products. Finally, the PCR amplification products were quantified using a QuantiFluor™–ST blue fluorescence quantitative system (Promega, Madison, WI, USA). All sequences were performed on an Illumina PE250 platform.

Bioinformatics analysis: The relevant raw data were analyzed using QIIME (quantitative insights into microbial ecology) [49]. After quality control and filtering of low-quality sequences, the resulting valid sequences were clustered into operational taxonomic units (OUT), according to 97% similarity [50]. Based on the OTU cluster analysis results, alpha diversity, with indices of Ace, Chao1, Shannon, and Simpson, was used to reflect the abundance and diversity of microbial communities. The similarities and differences between groups (between samples) were analyzed based on the PCoA of UniFrac (Beta diversity analysis) [32]. The community structure of SE–SSOs before and after SPH treatment was statistically analyzed at the phylum, family, and genus levels. Then, LEfSe used LDA pairwise analysis (SE–SSOs versus SPH treatment for 12 h, SE–SSOs versus SPH treatment for 24 h) to identify bacterial populations that have significant differences in sample classification. Furthermore, PICRUSt was used to predict the functional changes of SE–SSOs after SPH treatment using COG analysis.

### 3.9. Statistic Analysis

Data were expressed as mean ± standard deviation (*n* = 3). One-way variance (ANOVA) and Tukey’s test were analyzed using SPSS^®^ software 19.0 (Chicago, IL, USA) to present significant differences among the mean values at the *p* ≤ 0.05.

## 4. Conclusions

SPH can inhibit SE–SSOs through membrane damage. Sequencing of 16S rDNA amplifiers revealed that SE–SSOs are mainly composed of two phyla of Firmicutes and Proteobacteria (with a total relative abundance of 99.9%). SPH treatment can significantly reduce the relative abundance of the conditional pathogenic bacterium Peptostreptococcaceae in SE–SSOs while promoting the growth of Enterococcaceae related to bacteriocin production. SPH has a significant inhibitory effect on the genus of *Paraclostridium*, while two genera of *Enterobacter* and *Enterococcus* became SSOs after SPH treatment. Our results confirmed that SPH treatment can change the bacteria structure of SE–SSOs, which might be related to the inhibition of cell-signal transduction between specific bacteria by SPH. SPH could be further used for the preservation of squid, with a broad development prospect. However, the specific regulatory pathways of SPH on SE–SSOs need to be further explored in subsequent experiments.

## Figures and Tables

**Table 1:** Hydrolysis pH and temperatures of different proteases used for preparation of shrimp by-product hydrolysates.

Proteases	Hydrolysis pH	Hydrolysis Temperature (°C)
Neutral protease	7.0	50
Flavourzyme	8.0	50
Trypsin	8.0	37
Alcalase	9.0	45
Pepsin	2.0	35

Note: Minced shrimp by-products were blended with distilled water at a controlled solid to liquid ratio of 1:2 (*w*:*v*).

**Table 2:** Alpha diversity index of the SE–SSOs after treated with SPH (*n* = 3).

Group	Ace Index	Chao1 Index	Shannon Index	Simpson Index	Coverage
CK	9.50 ± 2.12 ^a,b^	7.67 ± 1.15 ^a,b^	1.15 ± 0.04 ^b^	0.37 ± 0.01 ^a^	0.9999
SPH–12 h	5.67 ± 0.58 ^a^	5.67 ± 0.58 ^a^	0.88 ± 0.13 ^a^	0.46 ± 0.04 ^b^	1.0000
SPH–24 h	10.33 ± 1.53 ^b^	10.67 ± 1.53 ^b^	0.91 ± 0.05 ^a^	0.43 ± 0.00 ^a,b^	0.9999

Note: Different lowercase letters under the same index represented significant differences between the CK, SPH–12 h, and SPH–24 h groups (*p* < 0.05).

## Data Availability

Data will be made available on request.

## Supplementary Materials

The following supporting information can be downloaded at: https://www.mdpi.com/article/10.3390/molecules28104105/s1, Figure S1: Antibacterial activity of SPH on SE-SSOs measured by agar well diffusion method.
