# Supplementary figures and images for: Antibacterial Effect of Shrimp By-Products Hydrolysate on Specific Spoilage Organisms of Squid
Source: Molecules. 2023 May 15;28(10):4105. doi: 10.3390/molecules28104105 (PMC10221855; doi:10.3390/molecules28104105)

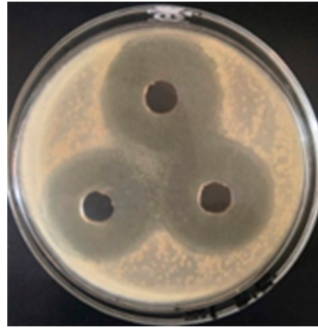

Figure S1 Antibacterial activity of SPH on SE-SSOs measured by agar well diffusion method.

Supplement: Supplementary file 1 [file molecules-28-04105-s001.zip › molecules-2336593-supplementary.pdf]
